# Selective Serotonin Reuptake Inhibitor Treatment in Adolescence and Subsequent Risk of Nonaffective Psychosis: A Quasi‐Experimental Study

**DOI:** 10.1111/acps.70098

**Published:** 2026-04-07

**Authors:** Ioanna Kougianou, Animesh Talukder, Kirstie O′ Hare, Colm Healy, Ian Kelleher

**Affiliations:** ^1^ Division of Psychiatry, Institute for Neuroscience and Cardiovascular Research University of Edinburgh Edinburgh UK; ^2^ Discipline of Psychiatry and Mental Health University of New South Wales Sydney New South Wales Australia; ^3^ School of Medicine University College Dublin Dublin Ireland; ^4^ Faculty of Medicine University of Oulu Oulu Finland; ^5^ St John of God Hospitaller Services Group Dublin Ireland

**Keywords:** depression, psychotic disorders, selective serotonin reuptake inhibitors

## Abstract

**Objective:**

Psychotic disorders are frequently preceded by common mental disorders (CMDs), like depression and anxiety. It remains unclear whether treating CMDs in adolescence can reduce subsequent psychosis risk. We applied quasi‐experimental methods to national register data to assess whether a causal relationship exists between selective serotonin reuptake inhibitor (SSRI) treatment in adolescence and subsequent risk of psychosis.

**Methods:**

Using the secure anonymized information linkage databank, we identified individuals living in Wales (born between 1991 and 1998) with depression who attended Child and Adolescent Mental Health Services. We employed an instrumental variable (IV) analysis, using regional variation in SSRI prescribing practice to estimate the causal effect of cumulative SSRI treatment on psychosis risk, applying two‐stage least squares and IV probit models.

**Results:**

Our cohort included *n* = 6615 individuals with adolescent depression. We found no evidence that cumulative SSRI prescription influenced the psychosis risk across intervention windows (1 year: *β*: 0.114, CI: −0.049, 0.276; 2 years: *β* 0.062, CI: −0.114, 0.239; 3 years: *β* 0.021, CI: −0.125, 0.168).

**Conclusion:**

This quasi‐experimental study found no evidence of a causal relationship between SSRI treatment in adolescence and later psychosis risk. Our findings do not support the hypothesis that SSRI treatment of CMDs in adolescence prevents later psychosis.

## Key Points

- Significant Outcomes
  ○ Using quasi‐experimental methods in a national cohort of adolescents, this study found a lack of causal evidence to support the hypothesis that antidepressant treatment in adolescence reduces risk of later psychotic disorder.
  ○ The findings of this study suggest that previously reported associations between antidepressant treatment and reduced psychosis risk were likely a result of confounding by indication, rather than reflecting a true causal effect.
  ○ More broadly, the study adds to the emerging literature challenging the “intervention as prevention” hypothesis, highlighting the need for robust causal inference methods to test assumptions about the long‐term impacts of youth mental health treatments.
- Limitations
  ○ While the analytic approach represents a robust causal inference design, it cannot fully exclude the possibility of residual confounding.
  ○ Treatment exposure was based on prescription records; it was not possible to measure treatment adherence.
  ○ The study is limited to pharmacological treatment—psychological treatments should be more carefully documented in healthcare registers to allow similar methods to be applied to them.

## 1 Introduction

Psychotic disorders are among the leading causes of disability worldwide [1]. Early identification of individuals at risk for psychosis has been a major focus of psychiatric research, with the goal of early intervention and prevention [2].

National cohort studies following young people from childhood into adulthood have shown that as many as half of all individuals in the population who are diagnosed with psychosis by the age of 30 years had at some point attended child and adolescent mental health services (CAMHS) [3, 4], most frequently for treatment of depressive and anxiety disorders [3]. Researchers have proposed that selective serotonin reuptake inhibitor (SSRI) medication treatment may reduce psychosis risk through a number of mechanisms, including changes in brain morphology [5], modulation of mitochondrial processes [6], alteration of the neurochemical pathways involved in the stress response, and reduction of faulty appraisals of the early psychotic experiences [7].

A number of observational studies in individuals at clinical high risk (CHR) for psychosis have suggested a possible beneficial effect of antidepressant treatment on reducing later psychosis risk [7, 8, 9]. In one naturalistic treatment study, for example, it was found that none of the CHR adolescents treated with antidepressants were diagnosed with psychosis, compared to 43% of CHR adolescents treated with antipsychotic treatment [8]. Similarly, in another naturalistic study, researchers found that only 8% of patients treated with antidepressants were diagnosed with psychosis, compared to 29% of patients treated with antipsychotics [7].

Observational studies are subject to significant bias, including, notably, confounding by indication. That is, key factors that might drive treatment assignment (in this case antidepressant vs. antipsychotic treatment) may also be associated with the outcome of interest (psychosis risk) [10]. The choice to treat CHR patients with antipsychotics versus antidepressants in naturalistic settings is not random—it is likely that factors related to psychosis risk (such as more severe illness or the presence of concerning psychotic symptoms) may have been related to both the decision to prescribe antipsychotics (versus antidepressants) and the risk of psychosis.

Even though randomized‐controlled trials (RCTs) are considered the gold‐standard for testing causal relationships, there are practical and ethical constraints, to running an RCT. RCTs typically have short follow‐up times, making it difficult to explore long‐term outcomes. In addition, it would not be considered ethical to withhold an effective treatment (i.e., antidepressant treatment for depression) to test for a hypothetical relationship with psychosis risk.

Observational data, including data from electronic health records (EHRs), allows for long‐term follow‐ups of individuals in a resource‐efficient way that reflects real‐world settings. Pretreatment characteristics that may influence treatment decisions and later outcomes, however, are typically not recorded in EHRs, making it difficult to assess the causal effects of treatments on health outcomes. In this type of scenario, quasi‐experimental methods, such as instrumental variable (IV) designs, can help to explore questions about causality between a treatment and a health outcome [11, 12]. IV analyses aim to address the issue of unmeasured confounding by using an exogenous source of variation in the treatment—the instrument [11, 12].

Prescribing practice has previously been shown to vary across different centers [13]. Therefore, one frequently used IV candidate is a preference‐based instrument which exploits the naturally occurring variation in patterns of prescribing and can be calculated at either individual level [14] or regional level [15, 16].

We applied an IV design to national registry data in order to robustly assess for a causal relationship between SSRI antidepressant treatment in patients with CAMHS contact and subsequent risk of psychosis. Just one study to date has investigated the relationship between SSRI treatment and psychosis risk in CAMHS. Using Finnish healthcare registry data, Healy et al. [17] recently leveraged regional variation in prescribing practice across Finnish hospital districts to investigate this causal question. In contrast to previous observational studies, which did not apply robust quasi‐experimental designs [2, 8], Healy et al. [17] found no evidence to support the hypothesis that SSRI treatment reduced risk of later psychosis. Given the importance of this research question, we aimed to conduct a robust replication of this study, using UK national registry data. Specifically, we applied an IV design to robustly assess for a causal relationship between SSRI treatment in CAMHS patients in Wales and subsequent psychosis risk when followed into adulthood, using regional variation in SSRI prescribing practice as our instrument.

## 2 Methods

### 2.1 Data Source

We used the Secure Anonymised Information Linkage (SAIL) databank [18, 19, 20, 21], which was developed at the Health Information Research Unit in the School of Medicine at Swansea University [20]. It contains anonymized, routinely collected data for the population of Wales across a range of datasets (social care, health care, and administrative) accessible via a trusted research environment [18]. SAIL data is anonymized using a split‐file approach to maintain confidentiality [18, 20].

The datasets in SAIL databank used in the current study were [1]; Welsh Demographic Service Dataset (WDSD), which includes administrative information about individuals that utilize National Healthcare Services (NHS) in Wales (available from 1990 to end of study) [2]; Welsh Longitudinal General Practice (WLGP) dataset, a primary care dataset, covering ~83% of the population of Wales (available from 2000 to end of study). The WLGP uses a hierarchical coding system, known as Read codes, to record clinical information like symptoms, diagnoses, and medications [3]; Patient Episode Dataset for Wales (PEDW), containing records of hospital inpatient admission in NHS hospitals in Wales (available from 1995 to end of study) [4]; Outpatient Database for Wales (OPDW), which includes outpatient NHS hospital appointments in Wales (available from 2004 to end of study) [5]; National Community Child Health Database (NCCH), which includes birth registration and monitoring of child immunizations and health examinations (available from 1989 to end of study).

SAIL's information governance review panel granted approval to conduct this research (IGRP Number 1635).

### 2.2 Sample

**FIGURE 1:**
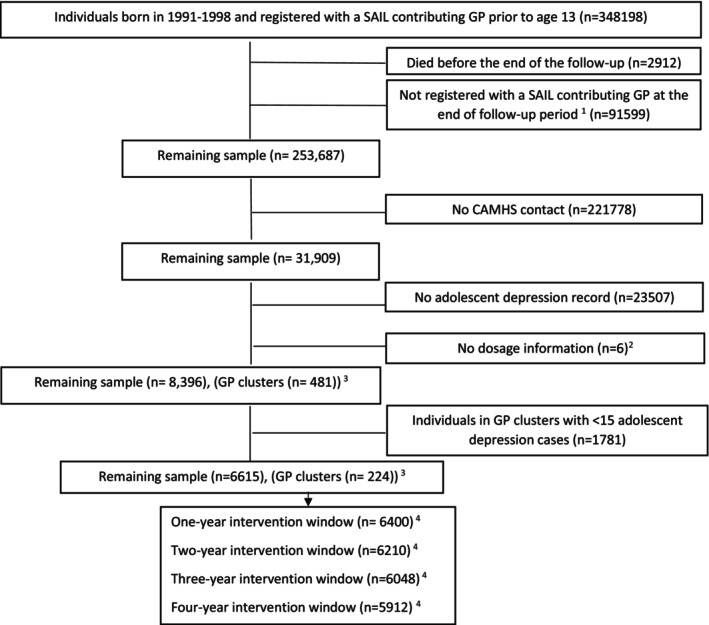
Flowchart for the study sample. ^1^End of follow‐up period (November 2023). ^2^We removed individuals if they had a record of the following SSRI prescription read codes: da4.., da5.., da9.. .^3^Here, along with the number of individuals within the study sample, we are reporting the number of general practice (GP) clusters. ^4^Sample excludes the individuals who developed the outcome before the end of each intervention window. CAMHS, Child and Adolescent Mental Health Service; GP, general practice, SSRI, Selective Serotonin Reuptake Inhibitor.

The sample included individuals who met the following criteria [1]: born between 1991 and 1998 (inclusive) [2], registered with a SAIL contributing general practice (GP) prior to age 13 years [3], one CAMHS contact prior to age 18 years [4], a record of depression diagnosis or depressive symptom in adolescence (13–17 years old; inclusive) [5], registered with a SAIL contributing GP at the end of follow‐up period and [6] not died prior to end of follow‐up (November 2023). The study flowchart can be found in Figure 1.

### 2.3 Contact With CAMHS

Child and adolescent psychiatric care in Wales is delivered almost exclusively within NHS; private provision of child and adolescent psychiatry is minimal in Wales and was not included in this study. CAMHS contact was identified from the following datasets: WLGP, PEDW, and OPDW. In WLGP, CAMHS contact was identified using Read Codes which indicated contact with a specialist mental health service prior to age 18 years. The code list was adapted from Joseph et al. [22] (Table S1.1). In PEDW, CAMHS contact was defined as any admission for any primary diagnosis of a mental disorder (International Classification of Diseases (ICD)‐10 codes F00‐99) beginning prior to age 18 years, or an admission where the speciality code attached to the record was a relevant psychiatry code (Table S1.1), beginning prior to age 18 years. Finally, in OPDW, CAMHS contact was defined as any appointments occurring prior to age 18 years where the speciality code attached to the record was a relevant psychiatry code (Table S1.1). A variable was constructed to indicate the earliest CAMHS contact date (among the three datasets) at any point prior to age 18 years.

### 2.4 Indication for SSRI Treatment

Inclusion criteria for the study included a medical record of depression diagnosis or depressive symptom in adolescence (13–17 years old; inclusive). Depressive symptoms were considered as an indication for SSRI prescription alongside depression diagnoses, as it has been previously shown that there is an increasing trend in recent years of recording symptoms rather than diagnoses of depression in young people (< 18 years of age) in primary care records in Wales [23]. Furthermore, the use of both symptom and diagnosis codes has been shown to have a good specificity and positive predictive value for detecting depression cases [23]. Depression diagnosis and symptom were identified from the WLGP dataset. The code list was adapted from John et al. [24] and reviewed by a child and adolescent psychiatrist (IK) (Table S1.2). Depression diagnoses were also identified in the PEDW dataset (F32‐34, ICD‐10 codes) (Table S1.2).

### 2.5 Intervention Windows

We constructed intervention windows which began on the date of indication—earliest record of adolescent depression for each individual. These intervention windows counted the amount of SSRI treatment prescribed within the specified time: 1‐, 2‐, 3‐, and 4‐year intervention windows.

### 2.6 SSRI Treatment

We examined the following SSRIs: fluoxetine, sertraline, and citalopram. SSRI prescription was identified from the WLGP dataset using Read codes, and the codelist was adapted from John et al. [24] (Table S1.3).

SSRI treatments were standardized based on the World Health Organization's (WHO) daily defined dose (DDD) from the anatomical therapeutic code classification system [25]. This standardization allowed us to examine the cumulative amount of SSRI treatment received across all SSRIs. For each SSRI, the DDDs were: fluoxetine (N06AB03) 20 mg, sertraline (N06AB06) 50 mg, citalopram (N06AB04) 20 mg [25]. For each individual, SSRI treatment was defined as the cumulative DDDs within each intervention window. The DDD‐standardized SSRI treatment was also standardized by time within the intervention window, so that a score of the 4‐year intervention window, corresponded to 4 years of sustained SSRI treatment at the DDD.

### 2.7 IV Approach

We defined regional level prescribing preference as variation in prescribing practices across GPs, with GP being the region/cluster. The instrument was calculated as the average number of DDDs given for all individuals (leave‐one‐out self‐exclusion) with depression within each GP cluster for each treatment window. To avoid unstable estimates of the instrument, we only included GP clusters with ≥ 15 adolescent depression cases.

### 2.8 Outcome

Nonaffective psychosis diagnoses were identified from the WLGP dataset using Read codes and the inpatient dataset, PEDW, according to ICD‐10 codes (F20–F29). Read codes were adapted from Abel et al. [26] (Table S1.4). The date of diagnosis was identified from the record with the earliest date between PEDW and WLGP. Subsequently, a binary indicator was created to denote the presence or absence of nonaffective psychosis. If cases of nonaffective psychosis occurred prior to the record of depression or the end of the treatment window, they were excluded from all analyses.

### 2.9 Confounders

We used a directed acyclic graph to display our causal assumptions (Figure S2.1). We adjusted for potential confounders at an individual level as an attempt to capture patient‐mix, including: sex, year of birth, low birth weight, winter birth, deprivation quintile, urbanicity, age at adolescent depression, year of adolescent depression, history of SSRI prescription, and history of inpatient CAMHS contact. Sex was derived from the WDSD dataset and coded as female or male. Year of birth variable was identified from the WDSD dataset. Low birthweight was defined as birth weight < 2500 g (from NCCH). An indicator was created for winter birth status if the month of birth occurred (as recorded in WDSD) in December, January, or February. Using the 2014 version of the Welsh Index of Multiple Deprivation (WIMD) [27], socioeconomic deprivation was identified from each individual's earliest recorded area code available in the WDSD (WIMD quintiles, 1–5, with 1 being the most deprived). Urbanicity was identified using the 2011 rural–urban classification [28] of each individual's earliest area code record available in the WDSD dataset, and used to create an indicator for urban versus rural location. Age and year of healthcare contact for depression variables were calculated using date of event from WLGP dataset. A binary indicator to denote whether an individual had a history of an SSRI prescription (before their earliest record of adolescent depression) was also constructed. A binary indicator was constructed for history of inpatient CAMHS contact, defined as having contact with inpatient CAMHS (from PEDW) at any time point before the earliest depression indication in adolescence.

We also included confounders at the cluster level to capture regional level patient‐mix, including: percentage of CAMHS contact per GP, percentage of individuals living in urban areas per GP, percentage of individuals in each deprivation quintile per GP, and population size registered per GP. The percentage of CAMHS contact per GP was defined as the proportion of individuals who had any contact with CAMHS service (birth up to and including 17 years of age) within the same GP divided by the number of people registered to that same GP. The percentage of individuals living in urban areas per GP was defined as the proportion of individuals living in an urban residence within the GP divided by the number of people registered to that same GP. The percentage of individuals in each deprivation quintile was defined as the proportion of individuals who belonged to each WIMD quintile within the GP divided by the number of people registered to that same GP.

### 2.10 Statistical Analyses

We report descriptive characteristics of our cohort in terms of baseline covariates and clinical characteristics.

Logistic regression was used to examine odds ratios for the relationship between the cumulative SSRI prescription and nonaffective psychosis risk in individuals with adolescent depression, controlling for observed confounders.

Our IV approach was in line with the approach taken in Healy et al. [17] and Widding–Havneraas et al. [29]. Local average treatment effect (LATE) was the causal estimand and provides information for individuals who are prescribed an SSRI treatment due to the instrument and no SSRI treatment assignment due to the instrument [30]. LATE was calculated using a two‐stage least squares (2SLS) estimator. To fit the 2SLS model, the ivreg() function from the “AER” package was used [31]. Since the outcome is binary, we repeated the analysis using a nonlinear model for robustness, the IV Probit, which also estimates LATE. Probit models restrict the predicted values to be bound between zero and one [32]. The IV probit model was used to produce average marginal effects which can be interpreted as risk differences in the probability of the outcome [32]. Robust standard errors (SE) were employed to account for heteroscedasticity, clustered at the cluster level to account for within‐cluster correlation and calculated for all analyses using the “lmtest” and “sandwich” packages [33, 34]. The same confounders were adjusted for in the IV model as in the conventional logistic regression analysis. The resulting coefficient estimates were interpreted as the percentage point change in the probability of the outcome for each additional daily defined dose of SSRI treatment per day, on average for the duration of the intervention window.

In IV analysis, the following assumptions should be satisfied: relevance, exclusion, independence, and monotonicity. Please refer to Supporting Information 3 here, which helpfully details these assumptions.

SQL Db2 was used to interrogate data tables within the SAIL databank and all analyses were carried out using R studio (version 4.3.3). No artificial intelligence was used.

### 2.11 Sensitivity Analysis

We performed a sensitivity analysis, where we repeated our main analyses for each SSRI type independently: fluoxetine‐only, citalopram‐only, and sertraline‐only.

## 3 Results

### 3.1 Descriptive Statistics

We identified 8396 individuals who had an adolescent depression, were born between 1991 and 1998, and had a CAMHS contact at some point in childhood or adolescence (Figure 1). After restricting to GP clusters that included 15 or more individuals with adolescent depression the remaining sample was 6615 individuals with 224 GP clusters (Figure 1; Supporting Information S4.1). The majority of the sample were female (*n* = 4788, 72.38%; Table 1) and resided in one of the two most deprived WIMD quintiles (*n* = 3926, 59.35%; Table 1). In the study population, 4168 individuals had an SSRI prescription at least once between their earliest depression and subsequent 4 years of intervention windows (median time of SSRI prescription was 250.5 days, Supporting Information S4.2), while 2447 did not have an SSRI prescription in that time period. The prevalence of nonaffective psychosis diagnoses (anytime from birth until the end of follow‐up) in the study population was 273 individuals (4.13%; Table 1), and most (*n* = 203; Table 1) of the diagnoses occurred in the group exposed to SSRI prescription (maximum follow‐up ages, 25–32 years old; Table S5.1).

**TABLE 1:** Socioeconomic, clinical, and demographic characteristics of those with adolescent depression with and without SSRI prescription.^a^

Characteristics		Individuals with adolescent depression (among 1991–1998 birth cohort with CAMHS contact)		
		Overall	Depression with SSRI prescription^b^	Depression without SSRI prescription^b^
*N*		6615^c^	4168	2447
Sex (*n* [%])	Female	4788 (72.38%)	3152 (75.62%)	1636 (66.86%)
	Male	1826 (27.6%)	1015 (24.35%)	811 (33.14%)
	Missing	1 (0.02%)	1 (0.02%)	0 (0%)
Welsh index multiple deprivation (*n* [%])	Quintile 1 (most deprived)	2337 (35.33%)	1513 (36.3%)	824 (33.67%)
	2	1589 (24.02%)	1009 (24.21%)	580 (23.7%)
	3	1050 (15.87%)	652 (15.64%)	398 (16.26%)
	4	798 (12.06%)	484 (11.61%)	314 (12.83%)
	5 (least deprived)	841 (12.71%)	510 (12.24%)	331 (13.53%)
Urban residence (*n* [%])		5142 (77.73%)	3266 (78.36%)	1876 (76.67%)
History of inpatient CAMHS contact (*n* [%])		275 (4.16%)	184 (4.41%)	91 (3.72%)
History of SSRI prescription (*n* [%])		366 (5.53%)	336 (8.06%)	30 (1.23%)
Birth weight status (*n* [%])	Low birth weight	716 (10.82%)	472 (11.32%)	244 (9.97%)
	Missing	28 (0.42%)	14 (0.34%)	14 (0.57%)
Winter birth status (*n* [%])		1569 (23.72%)	1009 (24.21%)	560 (22.89%)
Age at first depression (mean [SD])		16.21 (1.25)	16.46 (1.14)	15.79 (1.31)
Age at first SSRI^a^ prescription (mean [SD])		—	17.56 (1.54)	—
Outcome				
Nonaffective psychosis (*n* [%])		273 (4.13%)	203 (4.87%)	70 (2.86%)

Abbreviations: CAMHS, Child and Adolescent Mental Health Service; SD, standard deviation; SSRI: selective serotonin reuptake inhibitors.

^a^ SSRI: fluoxetine, sertraline and citalopram.

^b^ Individuals with or without SSRI prescription within the 4 years of intervention window since the earliest depression.

^c^ The cohort was restricted to general practice clusters with 15 or more individuals with adolescent depression.

### 3.2 Conventional Analysis

In the unadjusted model, which did not adjust for the potential confounders, we found no evidence of association between cumulative SSRI treatment in individuals with adolescent depression and nonaffective psychosis risk throughout all four intervention windows (Table 2). After adjustment for the potential confounders, we also did not observe any evidence of association between SSRI prescription for adolescent depression indication and nonaffective psychosis (Table 2).

**TABLE 2:** Logistic regression for the association between SSRI treatment and nonaffective psychosis risk in individuals with adolescent depression.

Exposure (standardized cumulative SSRI prescription)	Nonaffective psychosis	
	Adjusted^a^ [OR, 95% CI]	Unadjusted^b^ [OR, 95% CI]
1‐year intervention window	1.18 (0.962, 1.448)	1.114 (0.918, 1.353)
2‐years intervention window	1.157 (0.897, 1.492)	1.035 (0.807, 1.328)
3‐years intervention window	1.207 (0.925, 1.575)	1.044 (0.794, 1.374)
4‐years intervention window	1.238 (0.928, 1.652)	1.09 (0.813, 1.461)

*Note:* Both analyses accounted for cluster robust standard errors at the general practice level. Individuals who develop the outcome prior to the end of the treatment window were omitted from the analysis.

Abbreviations: CI, confidence interval; OR, odds ratio; SSRI, selective serotonin reuptake inhibitors.

^a^ We adjusted for the following confounders: individuals' sex, deprivation quintiles, year of birth, urban residence area, low birth weight status, age at earliest depression record, winter birth status, history of SSRI prescription, year of earliest depression record, history of inpatient CAMHS contact, population size of the general practices (GP) they were registered to, % of population per GP who belong to each of the five deprivation quintiles, % of population per GP with a CAMHS contact, and % of population per GP who are from urban area.

^b^ Unadjusted analysis excluded all the confounders described above.

### 3.3 IV Analyses

We observed considerable variation in terms of regional variation in SSRI prescribing practices for adolescent depression (Table S6.1). The strength of the instrument (relevance) was assessed using the partial *F*‐statistic in the first‐stage regression. We found that the instrument was associated with the treatment in the first three intervention windows (partial *F*‐statistic range: 13.153–18.984). From the 2SLS model, we found no evidence that cumulative SSRI treatment for adolescent depression alter the risk subsequent of nonaffective psychosis (Table 3). In line with the 2SLS model, from the IV probit we found no evidence of association between SSRI treatment and later psychosis risk (Table S7.1). Given the weak *F*‐statistic (< 10) from the first‐stage regression, we did not report the estimate from the fourth intervention window. Finally, when we repeated the IV analysis by SSRI type, we observed a non‐weak *F*‐statistic only in the 2‐ and 3‐year intervention window of fluoxetine. From our IV fluoxetine‐only subgroup analysis, fluoxetine treatment was not associated with reduced risk of subsequent psychosis (Supporting Information 8).

**TABLE 3:** IV approach investigating the relationship between provider's preference for SSRI prescribing across general practice clusters and nonaffective psychosis risk in individuals with adolescent depression using Two‐Stage Least Squares regression (2SLS).

Providers preference for SSRI prescribing across general practices (IV instruments)	Nonaffective psychosis
*1‐year intervention window*	
Number of outcomes	215
*F*‐statistic (first‐stage regression)	17.304
IV effect 2SLS [*β* coefficient, 95% CI]	0.114 (−0.049, 0.276)
*2‐years intervention window*	
Number of outcomes	190
*F*‐statistic (first‐stage regression)	18.984
IV effect 2SLS [*β* coefficient, 95% CI]	0.062 (−0.114, 0.239)
*3‐years intervention window*	
Number of outcomes	162
*F*‐statistic (first‐stage regression)	13.153
IV effect 2SLS [*β* coefficient, 95% CI]	0.021 (−0.125, 0.168)
*4‐years intervention wind*ow	
Number of outcomes	136
*F*‐statistic (first‐stage regression)	7.803

*Note:* We adjusted for the following confounder: individuals' sex, deprivation quintiles, year of birth, urban residence area, low birth weight status, age at earliest depression record, winter birth status, year of earliest depression record, history of SSRI prescription, history of inpatient CAMHS contact, population size of the general practices (GP) they were registered to, % of population per GP who belong to each of the five deprivation quintiles, % of population per GP with a CAMHS contact, and % of population per GP who are from urban area. Analysis accounted for cluster robust standard errors at the general practice level. Individuals who develop the outcome prior to the end of the treatment window were omitted from the analysis.

Abbreviations: 2SLS, Two‐Stage Least Squares regression; CI, confidence intervals; IV, instrumental variable; SSRI, selective serotonin reuptake inhibitors.

## 4 Discussion

Previous research has suggested that SSRI treatment may reduce the risk of psychosis via a number of mechanisms including morphological changes in the brain [5], biological processes [6, 7], and reduction in faulty appraisal of early psychotic experiences [7]. We tested this hypothesis using quasi‐experimental methods in national registry data. Using an IV approach, exploiting the natural regional variation in prescribing practices, we tested for long‐term causal effects of SSRI treatment on psychosis risk. We found no evidence that SSRI treatment had a causal effect on subsequent risk of psychosis.

Previous observational studies indicating a possible protective effect of antidepressants against psychosis were unable to take into account the possibility of confounding by indication [7, 8]. That is, in those studies, which involved naturalistic clinical settings and treatments, the decision to treat with an antidepressant versus an antipsychotic is likely to have been affected by factors that are also related to psychosis risk (e.g., antipsychotics being more likely to be prescribed when the treating psychiatrist suspects a psychotic disorder is emerging).

Our IV findings align with a recent study by Healy et al. [17], which also employed quasi‐experimental methods to investigate for a causal relationship between SSRI treatment and subsequent risk of psychosis. Similarly, that study found that SSRI treatment in adolescence was not causally associated with a reduced risk of subsequent psychotic disorder when followed to adulthood.

Beyond the specific implications of our findings with regard to the causal effect of SSRI treatment on psychosis risk, our findings add to broader questions about the idea of “intervention as prevention.” That is, the assumption that the treatment of common mental disorders in youth, such as depression and anxiety, will inherently reduce the risk of later more severe mental illnesses, like schizophrenia. There have been few studies to date to formally test the “intervention as prevention” hypothesis, but those that exist have found, at best, mixed results [35, 36, 37, 38]. While some studies have found evidence that treatment in childhood may lead to better outcomes later in childhood [36, 39], the limited studies that have followed young people into adulthood have typically not found beneficial effects [38].

In the Great Smoky Mountains study, for example, Copeland et al. [37] found that while the use of mental health services in childhood may result in reduced psychopathology in the short term, it did not predict improved mental health outcomes when these children were followed to adulthood. Our findings add to this emerging literature, suggesting that it would be premature to assume that interventions for common youth mental health problems, such as depression and anxiety, will necessarily reduce the risk of later more severe mental illnesses. This also points to the need for more of a research focus on interventions that might impact on longer‐term mental health trajectories, rather than focusing only on short‐term outcomes typical of RCTs.

Approximately 60% of our adolescent depression sample was from the two most deprived WIMD quintiles. This is in keeping with previous research demonstrating a clear socioeconomic gradient in referrals to mental health services [40, 41, 42].

### 4.1 Strengths and Limitations

We extensively examined the IV assumptions either directly (relevance) or through falsification tests and subject‐matter knowledge for the remaining assumptions, which, overall, supported the validity of the instrument. For relevance we found the instrument was associated with the treatment in the first three intervention windows. For independence, we observed that the instrument was weakly associated with history of SSRI treatment and the percentage of CAMHS contact within the GP. This suggests we cannot fully exclude any possible relationship between the instrument and unobserved confounders. We adjusted for a large number of potential confounders in our analyses and thus attempted to minimize any effect they might have on the instrument, exposure and outcome. For the exclusion restriction, we observed no evidence of association between the instrument and nonaffective psychosis, in support of the validity of the instrument. While the analytic approach represents a robust causal inference design, we cannot fully exclude the possibility of residual confounding.

We had an extended follow‐up time (age: 25–32), thus allowing sufficient time for the outcome to occur. According to a meta‐analysis by Solmi et al. [43] 48% of schizophrenia‐spectrum disorders have onset by age 25 years and 75% by age 34 years. Thus, we expect we have identified the majority of psychosis cases. While our sample did not extend beyond age 32 years, it is unlikely that adolescent SSRI treatment would have a uniquely protective effect on psychosis cases arising after age 32 years if it did not have a protective effect before age 32 years.

We did not have information on treatment adherence, though it is likely that individuals who filled more prescriptions ultimately took a greater amount of the medication. Future research should investigate psychological, as well as pharmacological treatments—this will require more data on psychological treatments being captured in register datasets. Our study only included patients treated through the NHS. Private provision for child and adolescent psychiatry in Wales, however, is minimal, meaning this is unlikely to have affected our results.

In conclusion, quasi‐experimental methods in a large national register dataset, we did not find evidence to support the hypothesis that SSRI treatment impacts on subsequent risk of psychosis. Previous research suggesting a protective effect of SSRIs likely has suffered from significant biases, in particular confounding by indication. More broadly, our findings highlight the need for more research to assess the “intervention as prevention” hypothesis, using robust methods focused on examining the effect of treatments not just on short‐term outcomes but on long‐term trajectories.

## Author Contributions

**Ioanna Kougianou:** formal analysis, visualization, writing – original draft, writing – review and editing, data curation, software methodology, administration. **Animesh Talukder:** writing – review and editing, data curation methodology. **Kirstie O′ Hare:** writing – review and editing, data curation, software, methodology administration. **Colm Healy:** writing – review and editing, methodology, project administration, supervision. **Ian Kelleher:** conceptualization, funding acquisition, writing – review and editing, resources, supervision, project administration, methodology. All authors read and approved the final manuscript.

## Funding

This project was supported by awards to I.K. from the Health Research Board (ECSA‐2020‐005), the Academy of Medical Sciences (APR8\1005), and the UK Department for Business, Energy and Industrial Strategy. The funding source has no role in relation to the study design, collection, analysis, and interpretation of data, writing of the report, and decision to submit the article for publication.

## Conflicts of Interest

The authors declare no conflicts of interest.

## Supporting information


**Table S1:1.** Clinical codes used to identify child and adolescent mental health services (CAMHS) contact.
**Table S1:2.** Clinical codes to identify diagnosis and symptoms of depression.
**Table S1:3.** Clinical codes to identify selective serotonin reuptake inhibitor prescription.
**Table S1:4.** Clinical codes to identify nonaffective psychosis.
**Figure S2:1.** Directed acyclic graph (DAG).
**Figure S3:1.** (A–C) Balance plots showing the association between the instruments (1–3 years of provider's preference for SSRI prescribing across general practice clusters) and the confounders in those with adolescent depression.
**Figure S3:2.** Balance plot displaying the standardized mean difference in the control population.
**Table S3:1.** Association between instrument and nonaffective psychosis risk in a matched control population using ordinary least squares (OLS) regression.
**Table S3:2.** First‐stage regression across different strata of confounders in those with adolescent depression.
**Table S5:1.** Study cohort.
**Table S6:1.** Regional level variation in SSRI prescribing patterns across general practice clusters based on the standardized SSRI cumulative treatment in those with adolescent depression.
**Table S7:1.** IV Probit model with average marginal effects reported.
**Table S8:1.** Association between the IVs (over the course of different treatment windows) and cumulative prescription of sertraline or citalopram.
**Table S8:2.** 1991–1998 birth cohort clinical and demographic characteristics in those with adolescent depression.
**Table S8:3.** Variability in provider's preference for fluoxetine prescribing across general practice based on the standardized fluoxetine treatment in those with adolescent depression.
**Table S8:4.** Instrumental variable approach investigating the relationship between provider's preference for fluoxetine prescribing and nonaffective psychosis risk in those with adolescent depression using two‐stage least squares regression (2SLS).

## Data Availability

Access to SAIL data is available on application to the SAIL Databank via their usage governance process (www.saildatabank.com).
