# Supplementary material for: Selective Serotonin Reuptake Inhibitor Treatment in Adolescence and Subsequent Risk of Nonaffective Psychosis: A Quasi‐Experimental Study
Source: Acta Psychiatr Scand. 2026 Apr 7;154(2):153–62. doi: 10.1111/acps.70098 (PMC13327166; doi:10.1111/acps.70098)
Supplement: Supplementary file 1 — Table S1:1. Clinical codes used to identify child and adolescent mental health services (CAMHS) contact. Table S1:2. Clinical codes to identify diagnosis and symptoms of depression. Table S1:3. Clinical codes to identify selective serotonin reuptake inhibitor prescription. Table S1:4. Clinical codes to identify nonaffective psychosis. Figure S2:1. Directed acyclic graph (DAG). Figure S3:1. (A–C) Balance plots showing the association between the instruments (1–3 years of provider's preference for SSRI prescribing across general practice clusters) and the confounders in those with adolescent depression. Figure S3:2. Balance plot displaying the standardized mean difference in the control population. Table S3:1. Association between instrument and nonaffective psychosis risk in a matched control population using ordinary least squares (OLS) regression. Table S3:2. First‐stage regression across different strata of confounders in those with adolescent depression. Table S5:1. Study cohort. Table S6:1. Regional level variation in SSRI prescribing patterns across general practice clusters based on the standardized SSRI cumulative treatment in those with adolescent depression. Table S7:1. IV Probit model with average marginal effects reported. Table S8:1. Association between the IVs (over the course of different treatment windows) and cumulative prescription of sertraline or citalopram. Table S8:2. 1991–1998 birth cohort clinical and demographic characteristics in those with adolescent depression. Table S8:3. Variability in provider's preference for fluoxetine prescribing across general practice based on the standardized fluoxetine treatment in those with adolescent depression. Table S8:4. Instrumental variable approach investigating the relationship between provider's preference for fluoxetine prescribing and nonaffective psychosis risk in those with adolescent depression using two‐stage least squares regression (2SLS). [file ACPS-154-153-s001.docx]

**Supplementary material**

Contents

[**Supplementary 1.** Codelists 1](#_Toc225502812)

[**Supplementary 2:** Directed Acyclic Graph (DAG) 10](#_Toc225502813)

[**Supplementary 3:** Testing the assumptions of instrumental variable (IV) analysis 11](#_Toc225502814)

[**Supplementary 4** 16](#_Toc225502815)

[**Supplementary 5:** Study cohort timeline 16](#_Toc225502816)

[**Supplementary 6:** 17](#_Toc225502817)

[**Supplementary 7**: IV probit model with average marginal effects 17](#_Toc225502818)

[**Supplementary 8:** Subgroup analyses 18](#_Toc225502819)

[**References:** 21](#_Toc225502820)

### **Supplementary 1.** Codelists

**Table S1.1.** Clinical Codes used to identify Child and Adolescent Mental Health Services (CAMHS) contact

| **Psychiatry Specialty Codes** | |
| --- | --- |
| **Code** | **Description** |
| 710 | Mental Illness |
| 711 | Child & Adolescent Psychiatry |
| 712 | Forensic Psychiatry |
| 720 | Eating Disorders |
| 721 | Addiction Services |
| 722 | Liaison Psychiatry |
| 723 | Psychiatric Intensive Care |
| 724 | Perinatal Psychiatry |
| 725 | Mental Health Recovery and Rehabilitation Service |
| 726 | Mental Health Dual Diagnosis Service |
| 727 | Dementia Assessment Service |
| **Read Codes** | |
| **Code** | **Description** |
| 8HM9. | listed for psychiatric admissn |
| 8HlB. | urgent referral to psychiatrist |
| ZL1B2 | under care of forensic psychiatrist |
| 8A2.. | psychiatric monitoring |
| ZL62E | referral to psychiatric nurse |
| 8H7A. | refer to mental health worker |
| 8A21. | psychiatric observation |
| 8HBK. | mental health triage nurse follow up |
| 8CM9. | mental health cpa contingency plan available |
| 9NJb. | in-house psychiatry first appointment |
| 7P22. | rehabilitation for psychiatric disorders |
| ZL231 | under care of community psychiatric nurse |
| 69F.. | mental health act examination |
| 8Hc1. | referral to mental health crisis team |
| ZL1B6 | under care of rehabilitation psychiatrist |
| ZL9D5 | seen by psychiatrist for mental handicap |
| 9bA.. | psychiatry |
| 9239 | fp22-in mental hospital |
| ZLE92 | discharge from forensic psychiatry service |
| 8Hc.. | referral to mental health team |
| ZL5B2 | referral to forensic psychiatrist |
| 9Nla0 | seen by child and adolescent psychiatrist |
| 9No0. | seen in child and adolescent psychiatry clinic |
| ZLD2b | discharge by child and adolescent psychiatrist |
| 8HHs. | referral to psychosis early intervention service |
| ZLD2f | discharge by psychiatrist for mental handicap |
| 9N2a. | seen by community psychiatric nurse |
| 9bA2. | child and adolescent psychiatry |
| 9Nk6. | seen in mental health clinic |
| 8H23. | admit psychiatric emergency |
| ZLD81 | discharge by community psychiatric nurse |
| 9N2v. | seen by primary care mental health gateway worker |
| 7P22z | rehabilitation for psychiatric disorders nos |
| ZLE91 | discharge from child and adolescent psychiatry service |
| 8HgB. | discharged by mental health primary care worker |
| ZL1B1 | under care of child and adolescent psychiatrist |
| 8CM2. | psychiatry care plan |
| 8HK9. | psychiatric d.v. requested |
| ZV692 | [v]psych patient - prev admitted other health care provider |
| ZL9D3 | seen by liaison psychiatrist |
| 8CQ1. | mental health care programme approach crisis plan |
| 9N2q. | seen by primary care graduate mental health worker |
| 8H34. | psychiatric day care |
| 8Hg9. | discharged from community mental health service |
| 8H38. | non-urgent psychiatric admisn. |
| ZV691 | [v]psych patient - prev admitted this health care provider |
| 9N5G4 | 111 contact disposition to mental health service |
| 8HHu. | referral to primary care mental health gateway worker |
| ZL5B5 | referral to psychiatrist for mental handicap |
| ZV690 | [v]psychiatric patient - not previously admitted |
| ZLE9. | discharge from psychiatry service |
| 9NNM. | under care of community psychiatric nurse |
| 8Hc0. | referral to community mental health team |
| 9bA0. | mental handicap (specialty) |
| 8HHv. | referral to primary care mental health graduate worker |
| 7P22y | other specified rehabilitation for psychiatric disorders |
| 8H4f. | referral to learning disabilities psychiatrist |
| ZLD7C | discharge by psychiatric nurse |
| 9Ng6. | independent mental capacity advocate instructed |
| ZL1B5 | under care of psychiatrist for mental handicap |
| 8H2T. | emergency voluntary psychiatric admission mental health act |
| 8H230 | emerg psychiatric admiss mha |
| 9N6h. | referral by mental health service |
| 8HVO. | private referral to psychiatrist |
| 9Nla. | seen by psychiatrist |
| 69FZ. | mental health act exam nos |
| ZV69211 | [v]psychiatric patient - previously admitted other district |
| 8HHR. | referral to child and adolescent psychiatry service |
| 9NN7. | under care of mental health team |
| ZLF22 | discharge from psychiatry day hospital |
| ZLD2g | discharge by rehabilitation psychiatrist |
| 8HlD. | referral to forensic psychiatrist |
| 8G131 | cbtp - cognitive behavioural therapy for psychosis |
| 8CQ0. | completion of mental health crisis plan |
| ZLD2c | discharge by forensic psychiatrist |
| ZL22E | under care of psychiatric nurse |
| 8HL9. | psychiatry d.v. done |
| 9N2z. | seen by child and adolescent mental health service |
| 8CY.. | mental health care programme approach |
| ZLD2a | discharge by psychiatrist |
| 8Ce7. | preferred place of care - mental health unit |
| 8H7B. | refer to community psych.nurse |
| ZLE93 | discharge from liaison psychiatry service |
| 8CM9. | mental health care programme approach contingency plan |
| 8H49. | psychiatric referral |
| ZL1B. | under care of psychiatrist |
| 8HgY. | discharge from child and adolescent mental health service |
| 9NNQ. | under care of hospital psychiatric team |
| ZL1B3 | under care of liaison psychiatrist |
| 9NN4. | has community mental health team key worker |
| 9N2E2 | seen by core psychiatry trainee doctor |
| 9Nla1 | seen by consultant psychiatrist |
| 9N2r. | seen by mental health triage nurse |
| ZL9D2 | seen by forensic psychiatrist |
| ZLA31 | seen by community psychiatric nurse |
| ZL5B4 | referral to rehabilitation psychiatrist |
| 9NN70 | under care of mental health in-reach team |
| ZL5B1 | referral to child psychiatrist |
| 8A2Z. | psychiatric monitoring nos |
| ZL5B3 | referral to liaison psychiatrist |
| 9N5F. | patient initiated encounter with mental health crisis team |
| ZV69. | [v]psychiatric paitient admission details |
| 8T21. | referral for mental health assessment |
| 8Hg7. | discharged from care of mental health triage nurse |
| Z922. | care planning under section 117 of mental health act 1983 |
| 9NI6. | psychiatric outreach clinic |
| 8HJ3. | psychiatric self-referral |
| ZL5B1 | referral to child and adolescent psychiatrist |
| 9NN5. | under care of psychiatrist |
| 9NJd. | in-house psychiatry follow-up appointment |
| ZL5B. | referral to psychiatrist |
| ZL9D1 | seen by child and adolescent psychiatrist |
| 9NlG. | seen by forensic psychiatrist |
| ZLA2E | seen by psychiatric nurse |
| ZLE94 | discharge from mental handicap psychiatry service |
| ZV691 | [v]psychiatric patient - previous admission this district |
| ZLE96 | discharge from rehabilitation psychiatry service |
| 8CQ.. | mental health crisis plan |
| ZL9D. | seen by psychiatrist |
| 8H4P. | referral to child psychiatrist |
| ZLD2d | discharge by liaison psychiatrist |
| 9bA1. | mental illness (specialty) |
| 8Hc2. | referral to primary care mental health team |
| 9bA3. | forensic psychiatry |
| 9NJc. | in-house psychiatry discharge |
| ZL9D6 | seen by rehabilitation psychiatrist |
| 9N1T. | seen in psychiatry clinic |

**Table S1.2.** Clinical codes to identify diagnosis and symptoms of depression

| **Depression diagnosis and symptoms** | |
| --- | --- |
| **Read codes** | |
| **Code** | **Description** |
| 1B17. | depressed |
| 1B1U. | symptoms of depression |
| 1BP.. | loss of interest |
| 1BP0. | loss of inter prev enjoy activ \| loss of interest in previously enjoyable activity |
| 1BQ.. | loss of capacity for enjoyment |
| 1BT.. | depressed mood |
| 1BU.. | loss of hope for the future |
| 2257 | o/e - depressed |
| E112. | Single major depressive episode |
| E1120 | Single major depressive episode, unspecified |
| E1121 | Single major depressive episode, mild |
| E1122 | Single major depressive episode, moderate |
| E1123 | Single major depressive episode, severe, without psychosis |
| E1124 | Single major depressive episode, severe, with psychosis |
| E1125 | Single major depressive episode, partial or unspecied remission |
| E1126 | Single major depressive episode, in full remission |
| E112z | Single major depressive episode NOS |
| E113. | Recurrent major depressive episode |
| E1130 | Recurrent major depressive episodes, unspecified |
| E1131 | Recurrent major depressive episodes, mild |
| E1132 | Recurrent major depressive episodes, moderate |
| E1133 | Recurrent major depressive episodes, severe, no psychosis |
| E1134 | Recurrent major depressive episodes, severe, with psychosis |
| E1135 | Recurrent major depressive episodes, partial/unspecified remission |
| E1136 | Recurrent major depressive episodes, in full remission |
| E1137 | Recurrent depression |
| E113z | Recurrent major depressive episode NOS |
| E118. | Seasonal affective disorder |
| E135. | Agitated depression |
| E204. | Neurotic depression reactive type |
| E291. | Prolonged depressive reaction |
| E2B.. | Depressive disorder NEC |
| E2B0. | Postviral depression |
| E2B1. | Chronic depression |
| Eu32. | [X]Depressive episode |
| Eu320 | [X]Mild depressive episode |
| Eu321 | [X]Moderate depressive episode |
| Eu322 | [X]Severe depressive episode without psychotic symptoms |
| Eu323 | Severe depressive episode with psychotic symptoms |
| Eu324 | [X]Mild depression |
| Eu325 | [x]major depression, mild |
| Eu326 | [x]major depression, moder sev \| [x]major depression, moderately severe |
| Eu327 | [x]maj dep, sev wthout psy sym \| [x]major depression, severe without psychotic symptoms |
| Eu328 | [x]Maj dep, sev with psyc symp |
| Eu329 | [X] Sin map dep ep sev ps ps rem |
| Eu32A | [X] Rec ma dep ep sev ps ps rem |
| Eu32B | [x]antenatal depression |
| Eu32y | [X]Other depressive episodes |
| Eu32z | [X]Depressive episode, unspecified |
| Eu33. | [X]Recurrent depressive disorder |
| Eu330 | [X]Recurrent depressive disorder, current episode mild |
| Eu331 | [X]Recurrent depressive disorder, current episode moderate |
| Eu332 | [X]Recurrent depressive disorder, current episode severe without psychotic symptoms |
| Eu333 | Recurrent depressive disorder, current episode with psychotic symptoms |
| Eu334 | [X]Recurrent depressive disorder, currently in remission |
| Eu33y | [X]Other recurrent depressive disorders |
| Eu33z | [X]Recurrent depressive disorder, unspecified |
| Eu341 | [X]Dysthymia |
| **International Classification of Diseases (ICD)-10** | |
| **Code** | **Description** |
| F32 | depressive episode |
| F32.0 | mild depressive episode |
| F32.1 | moderate depressive episode |
| F32.2 | severe depressive episode without psychotic symptoms |
| F32.3 | Severe depressive episode with psychotic symptoms |
| F32.8 | other depressive episodes |
| F32.9 | depressive episode, unspecified |
| F33 | recurrent depressive disorder |
| F33.0 | recurrent depressive disorder, current episode mild |
| F33.1 | recurrent depressive disorder, current episode moderate |
| F33.2 | recurrent depressive disorder, current episode severe without psychotic symptoms |
| F33.3 | recurrent depressive disorder, current episode severe with psychotic symptoms |
| F33.4 | recurrent depressive disorder, currently in remission |
| F33.8 | other recurrent depressive disorders |
| F33.9 | recurrent depressive disorder, unspecified |
| F34.1 | dysthymia |

**Table S1.3.** Clinical codes to identify selective serotonin reuptake inhibitor prescription

| **Selective Serotonin Re-uptake Inhibitor** | |
| --- | --- |
| **Read Codes** | |
| **Code** | **Description** |
| da41. | fluoxetine 20mg capsules |
| da42. | prozac 20mg capsules |
| da43. | fluoxetine 20mg/5ml oral liq \| fluoxetine 20mg/5ml oral liquid |
| da44. | prozac 20mg/5ml oral liquid |
| da45. | prozac 20mg capsules |
| da46. | fluoxetine 60mg capsules |
| da47. | prozac 60mg capsules |
| da48. | felicium 20mg capsules |
| da49. | oxactin 20mg capsules |
| da4A. | ranflutin 20mg capsules |
| da4B. | Prozit 20mg/5ml oral solution |
| da4C. | Prozep 20mg/5ml oral solution |
| da4D. | Olena 20mg dispersible tablets |
| da4E. | fluoxetine 20mg disp tabs \| fluoxetine 20mg dispersible tablets |
| da51. | sertraline 50mg tablets |
| da52. | sertraline 100mg tablets |
| da53. | lustral 50mg tablets |
| da54. | lustral 100mg tablets |
| da91. | citalopram 20mg tablets |
| da92. | cipramil 20mg tablet |
| da93. | citalopram 10mg tablets |
| da94. | cipramil 10mg tablet |
| da95. | citalopram 40mg tablets |
| da96. | cipramil 40mg tablet |
| da98. | paxoran 10mg tablet |
| da99. | paxoran 20mg tablet |
| da9A. | paxoran 40mg tablet |
| da9z. | citalopram 40mg/ml oral drops |

**Table S1.4.** Clinical codes to identify non-affective psychosis

| **Non-Affective Psychotic Disorders** | |
| --- | --- |
| **ICD Codes** | **Description** |
| F20 | Schizophrenia |
| F21 | Schizotypal disorder |
| F22 | Persistent delusional disorders |
| F23 | Acute and transient psychotic disorders |
| F24 | Induced delusional disorder |
| F25 | Schizoaffective disorders |
| F28 | Other nonorganic psychotic disorders |
| F29 | Unspecified nonorganic psychosis |
| **Read Codes** | **Description** |
| 1464. | H/O: schizophrenia |
| 146H. | H/O: psychosis |
| 212W. | Schizophrenia resolved |
| 212X. | Psychosis resolved |
| 285..11 | Psychotic condition, insight present |
| 286..11 | Poor insight into psychotic condition |
| E1… | Non-organic psychoses |
| E10.. | Schizophrenic disorders |
| E100. | Simple schizophrenia |
| E100.11 | Schizophrenia simplex |
| E100000 | Unspecified schizophrenia |
| E1001 | Subchronic schizophrenia |
| E1002 | Chronic schizophrenic |
| E1003 | Acute exacerbation of subchronic schizophrenia |
| E1004 | Acute exacerbation of chronic schizophrenia |
| E1005 | Schizophrenia in remission |
| E100z | Simple schizophrenia NOS |
| E101. | Hebephrenic schizophrenia |
| E1010 | Unspecified hebephrenic schizophrenia |
| E1014 | Acute exacerbation of chronic hebephrenic schizophrenia |
| E1015 | Hebephrenic schizophrenia in remission |
| E101z | Hebephrenic schizophrenia NOS |
| E102. | Catatonic schizophrenia |
| E1020 | Unspecified catatonic schizophrenia |
| E1021 | Subchronic catatonic schizophrenia |
| E1024 | Acute exacerbation of chronic catatonic schizophrenia |
| E1025 | Catatonic schizophrenia in remission |
| E102z | Catatonic schizophrenia NOS |
| E103. | Paranoid schizophrenia |
| E1030 | Unspecified paranoid schizophrenia |
| E1031 | Subchronic paranoid schizophrenia |
| E1032 | Chronic paranoid schizophrenia |
| E1033 | Acute exacerbation of subchronic paranoid schizophrenia |
| E1034 | Acute exacerbation of chronic paranoid schizophrenia |
| E1035 | Paranoid schizophrenia in remission |
| E103z | Paranoid schizophrenia NOS |
| E104. | Acute schizophrenic episode |
| E10y. | Other schizophrenia |
| E10y.11 | Cenesthopathic schizophrenia |
| E10y0 | Atypical schizophrenia |
| E10y1 | Coenesthopathic schizophrenia |
| E10yz | Other schizophrenia NOS |
| E10z. | Schizophrenia NOS |
| E121. | Chronic paranoid psychosis |
| E122. | Paraphrenia |
| E123. | Shared paranoid disorder |
| E123.11 | Folie a deux |
| E12z. | Paranoid psychosis NOS |
| E13.. | Other nonorganic psychoses |
| E13..11 | Reactive psychoses |
| E130.11 | Psychotic reactive depression |
| E133.11 | Bouffee delirante |
| E134. | Psychogenic paranoid psychosis |
| E13y. | Other reactive psychoses |
| E13y1 | Brief reactive psychosis |
| E13yz | Other reactive psychoses NOS |
| E13z. | Nonorganic psychosis NOS |
| E13z.11 | Psychotic episode NOS |
| E1y.. | Other specified non-organic psychoses |
| E1z.. | Non-organic psychosis NOS |
| Eu0z.12 | [X]Symptomatic psychosis NOS |
| Eu2.. | [X]Schizophrenia, schizotypal and delusional disorders |
| Eu20. | [X]Schizophrenia |
| Eu200 | [X]Paranoid schizophrenia |
| Eu20011 | [X]Paraphrenic schizophrenia |
| Eu201 | [X]Hebephrenic schizophrenia |
| Eu20111 | [X]Disorganised schizophrenia |
| Eu202 | [X]Catatonic schizophrenia |
| Eu20212 | [X]Schizophrenic catalepsy |
| Eu20213 | [X]Schizophrenic catatonia |
| Eu20214 | [X]Schizophrenic flexibilatis cerea |
| Eu203 | [X]Undifferentiated schizophrenia |
| Eu20311 | [X]Atypical schizophrenia |
| Eu205 | [X]Residual schizophrenia |
| Eu20511 | [X]Chronic undifferentiated schizophrenia |
| Eu206 | [X]Simple schizophrenia |
| Eu20y | [X]Other schizophrenia |
| Eu20y12 | [X]Schizophreniform disord NOS |
| Eu20y13 | [X]Schizophrenifrm psychos NOS |
| Eu20z | [X]Schizophrenia, unspecified |
| Eu21. | [X]Schizotypal disorder |
| Eu21.16 | [X]Pseudoneurotic schizophrenia |
| Eu21.17 | [X]Pseudopsychopathic schizophrenia |
| Eu22. | [X]Persistent delusional disorders |
| Eu220 | [X]Delusional disorder |
| Eu22011 | [X]Paranoid psychosis |
| Eu221 | [X]Delusional misidentification syndrome |
| Eu22111 | [X]Capgras syndrome |
| Eu222 | [X]Cotard syndrome |
| Eu22y | [X]Other persistent delusional disorders |
| Eu22z | [X]Persistent delusional disorder, unspecified |
| Eu23. | [X]Acute and transient psychotic disorders |
| Eu230 | [X]Acute polymorphic psychot disord without symp of schizoph |
| Eu23011 | [X]Bouffee delirante |
| Eu23012 | [X]Cycloid psychosis |
| Eu231 | [X]Acute polymorphic psychot disord with symp of schizophren |
| Eu23112 | [X]Cycloid psychosis with symptoms of schizophrenia |
| Eu232 | [X]Acute schizophrenia-like psychotic disorder |
| Eu23211 | [X]Brief schizophreniform disorder |
| Eu23212 | [X]Brief schizophrenifrm psych |
| Eu23214 | [X]Schizophrenic reaction |
| Eu233 | [X]Other acute predominantly delusional psychotic disorders |
| Eu23312 | [X]Psychogenic paranoid psychosis |
| Eu23y | [X]Other acute and transient psychotic disorders |
| Eu23z | [X]Acute and transient psychotic disorder, unspecified |
| Eu23z11 | [X]Brief reactive psychosis NOS |
| Eu23z12 | [X]Reactive psychosis |
| Eu24. | [X]Induced delusional disorder |
| Eu24.11 | [X]Folie a deux |
| Eu24.12 | [X]Induced paranoid disorder |
| Eu24.13 | [X]Induced psychotic disorder |
| Eu25012 | [X]Schizophreniform psychosis, manic type |
| Eu25112 | [X]Schizophreniform psychosis, depressive type |
| Eu26. | [X]Nonorganic psychosis in remission |
| Eu2y. | [X]Other nonorganic psychotic disorders |
| Eu2y.11 | [X]Chronic hallucinatory psychosis |
| Eu2z. | [X]Unspecified nonorganic psychosis |
| Eu2z.11 | [X]Psychosis NOS |
| ZV110 | [V]Personal history of schizophrenia |
| Eu251 | [X]Schizoaffective psychosis, depressive type |
| Eu250 | [X]Schizoaffective psychosis, manic type |
| E1074 | Acute exacerbation of chronic schizo-affective schizophrenia |
| Eu25. | [X]Schizoaffective disorders |
| 212T. | Psychosis, schizophrenia + bipolar affective disord resolved |
| Eu252 | [X]Mixed schizophrenic and affective psychosis |
| E1073 | Acute exacerbation subchronic schizo-affective schizophrenia |
| Eu25y | [X]Other schizoaffective disorders |
| E1070 | Unspecified schizo-affective schizophrenia |
| E1071 | Subchronic schizo-affective schizophrenia |
| Eu250 | [X]Schizoaffective disorder, manic type |
| Eu251 | [X]Schizoaffective disorder, depressive type |
| E1072 | Chronic schizo-affective schizophrenia |
| Eu25z | [X]Schizoaffective disorder, unspecified |
| E107. | Schizo-affective schizophrenia |
| Eu252 | [X]Schizoaffective disorder, mixed type |
| E1075 | Schizo-affective schizophrenia in remission |
| Eu25z | [X]Schizoaffective psychosis NOS |
| E107z | Schizo-affective schizophrenia NOS |

### **Supplementary 2:** Directed Acyclic Graph (DAG)


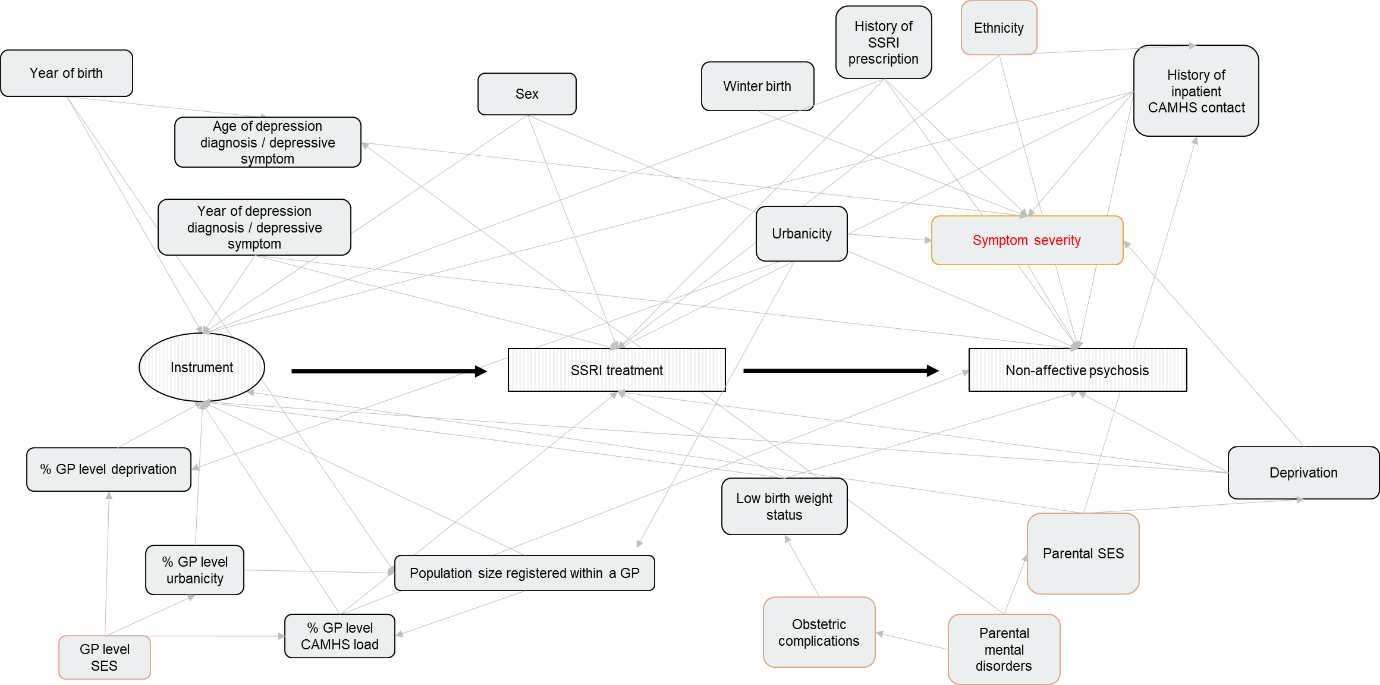
Figure S2.1. Directed Acyclic Graph (DAG). DAG denotes potential relationship between the instrument, SSRI treatment and non-affective psychosis. The observed and unobserved confounders at both the individual and regional cluster level, which were considered in this study, are included in the DAG. Unobserved confounders are also shown in the DAG as orange-outlined boxes and include: ethnicity, parental socioeconomic status, parental mental disorders, GP level SES, parental mental disorders and maternal obstetric complications.

**Note**: SSRI: Selective Serotonin Reuptake Inhibitors, SES: socioeconomic status, GP: general practice

### **Supplementary 3:** Testing the assumptions of instrumental variable (IV) analysis

IV analysis requires some assumptions to be satisfied for a variable to be a valid instrument and these are the following: (1) relevance, (2) independence, (3) exclusion restriction (1, 2). For the identification of the causal effect, the local average treatment effect, an additional assumption is required aside from those that should be met for a valid instrument, referred as (4) monotonicity(2).

Relevance is the first and only directly testable assumptions of the IV approach (3). The remaining assumptions ((2)-(4)) are not directly testable, but falsification tests can be employed to explore if there is evidence that they do not hold. We utilized linear regression (Ordinary Least Squares) models and clustered robust standard errors at the general practice (GP) level across models. Assumptions were tested in line with Healy *et al.,* (4).

**3.1 Relevance**

Relevance indicated that the instrument must be associated with the treatment (3) i.e., regional level of prescriber’s preference for SSRI associated with SSRI treatment. Relevance was empirically tested using first-stage regression, while adjusting for confounders (see methods; 2.9 confounders), and extracting the partial F-statistic. As a rule of thumb, an instrument is considered not weak when partial F-statistic is >10 (5). Here, we found that the partial F-statistic met the rule of thumb in the first three intervention windows (partial F-statistic range: 13.153 – 18.984, Table 3.).

**3.2 Independence**

Independence assumption entails that instrument is as-if random i.e., free from unobserved confounders. This assumption was assessed through falsification testing. We examine the relationship between the instrument with the observed confounders. Presence of an association between the instrument with the measured confounders would reduce the confidence that the analysis is entirely free from unmeasured confounding. Here, we tested assumption by running a multivariable regression with all the potential confounders of our study (see methods; 2.9 confounders) on the instruments. Balance plots were used for visualisation of the association between the instrument and the confounders over the various intervention windows (Figure S4.1 (A-C)). In one year and two-year intervention windows, the instrument was found to be associated with the history of SSRI prescription. In the three year intervention window, the instrument was found to be associated with the history of SSRI prescription and the percentage (%) of population with CAMHS contact per GP practice.

**Figure S3.1. (A-C).** Balance plots showing the association between the instruments (one – three years of provider’s preference for SSRI prescribing across general practice clusters) and the confounders in those with adolescent depression

1. **One year intervention window**

**
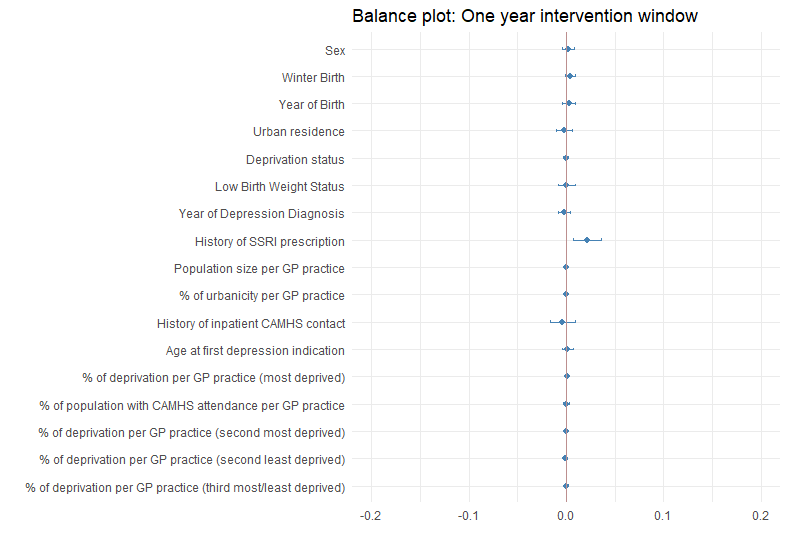
**

1. **Two years intervention window**

**
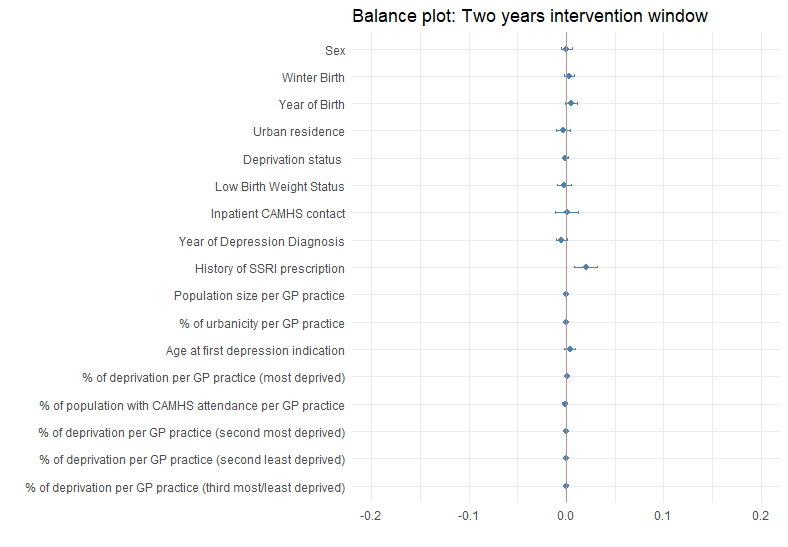
**

1. **Three years intervention window**

**
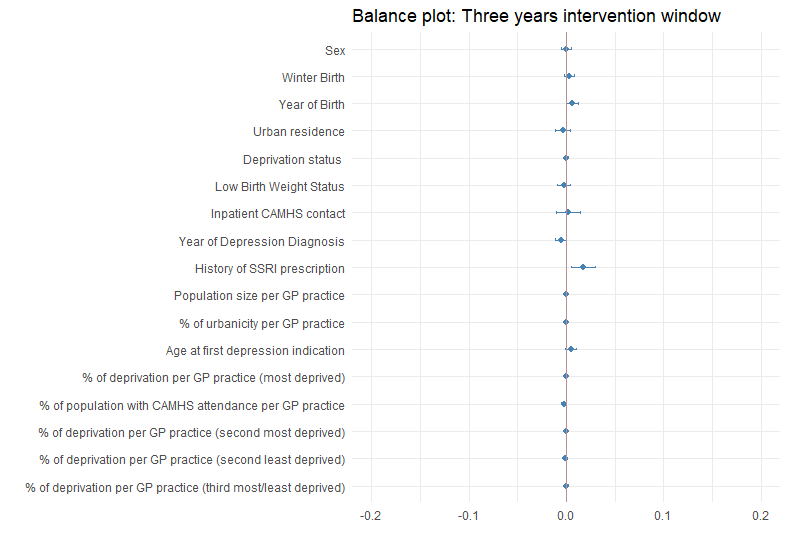
**

**3.4 Exclusion restriction**

Exclusion restriction assumes that the instrument should not affect the outcome independently, but other through the treatment (3). Our falsification tested exclusion restriction by examining the relationship between the instrument and non-affective psychosis in those who were not likely to have received any SSRI medication. To do this we matched (5:1 ratio) a sample of health control who had not been to CAMHS and had no SSRI prescription to our adolescent depression sample. We used nearest-neighbour method with the study sample on the following covariates: sex, deprivation quintiles, year of birth, urban residence area, low birth weight status and winter birth status and exactly matched on the general practice they were registered to. The balance between the two samples was tested descriptively to explore how well the matching is performed. In Figure S4.2. the balance plot displays the standardized mean difference in the control population prior and post matching. We used linear regression to test the relationship between the average GP-level SSRI prescribing propensity with non-affective psychosis in those who had not received SSRI medication (reduced form IV analysis). Here we found that there was no evidence of association between the instrument and the non-affective psychosis over the three intervention windows in the reduced form analysis, indicating that the instrument might only affect psychosis through exposure to SSRI treatment (Table S4.1).

**Figure S3.2.** Balance plot displaying the standardized mean difference in the control population


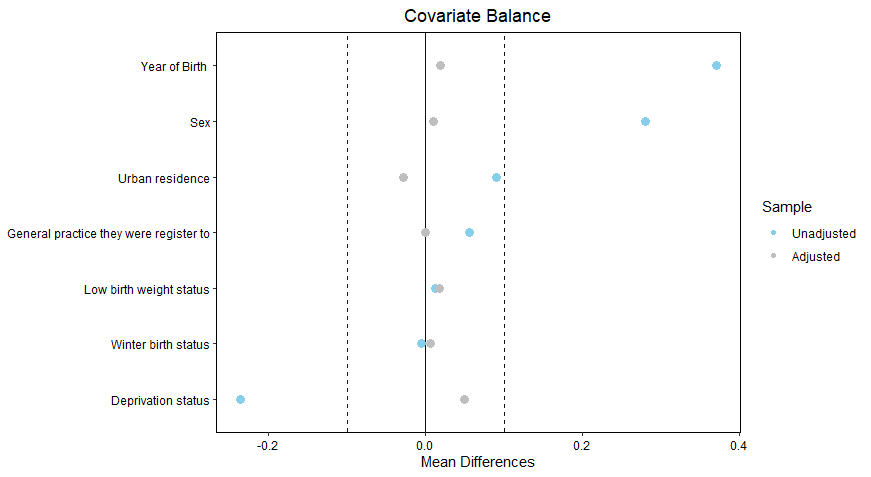


**Note:** We matched the control population on the following covariates: individuals’ sex, deprivation quintiles, year of birth, urban residence area, low birth weight status, winter birth status and exactly matched on the general practices (GP) they were registered to.

**Table S3.1.** Association between instrument and non-affective psychosis risk in a matched control population using Ordinary Least Squares (OLS) regression

| **Providers preference for SSRI prescribing**  **across general practice clusters**  **(IV instruments)** | **Non-affective psychosis** |
| --- | --- |
|  | *β* **coefficient (95% CI)** |
| One year | -0.002 (-0.005, 0.002) |
| Two years | -0.002 (-0.005, 0.002) |
| Three years | -0.001 (-0.005, 0.003) |

**Note:** We matched the control population on the following covariates: individuals’ sex, deprivation quintiles, year of birth, urban residence area, low birth weight status, winter birth status and exactly matched on the general practices (GP) they were registered to. Analysis accounted for cluster robust standard errors at the general practice level. Analysis was adjusted for the following confounders: individuals’ sex, deprivation quintiles, year of birth, urban residence area, low birth weight status. Analysis accounted for cluster robust standard errors at the general practice level.

SSRI: Selective Serotonin Reuptake Inhibitors, IV: instrumental variable, CI: confidence intervals

**3.5 Monotonicity**

The assumption of monotonicity refers to a subpopulation of individuals where their treatment assignment would be affected by changes in the instrument (6). In our study where the instrument is regional level prescriber’s preference for SSRI prescription, monotonicity would be described as: there would be no individuals treated with SSRI if they were assigned to a prescriber with low preference for SSRI treatment and no individuals not treated with SSRI if they were assigned to a prescriber with high preference for SSRI treatment. Here, this assumption can be falsified by examining the direction (first-stage regression *β* coefficient) between instrument and SSRI prescription by a series of stratification tests for each of the potential confounders.

**Table S3.2.** First stage regression across different strata of confounders in those with adolescent depression

|  |  | **Providers’ preference for SSRI prescribing across general practice clusters**  **(IV instruments)** | | |
| --- | --- | --- | --- | --- |
|  |  | **One year** | **Two years** | **Three years** |
|  |  | **First-stage *β* coefficient (95% CI)** | | |
| **Sex** | Male (n=1826) | 0.217 (0.063, 0.371) | 0.200 (0.097, 0.303) | 0.156 (0.053, 0.260) |
|  | Female (n=4788) | 0.207 (0.082, 0.333) | 0.224 (0.106, 0.343) | 0.263 (0.135, 0.392) |
| **Urban**  **residence** | Urban (n=5142) | 0.204 (0.033, 0.375) | 0.204 (0.021, 0.387) | 0.231 (0.037, 0.425) |
|  | Non-Urban (n=1473) | 0.136 ( -0.067, 0.338) | 0.165 (-0.045, 0.375) | 0.103 (-0.061, 0.267) |
| **Age at first depression** | 13-15(n=2580) | 0.204 (0.054, 0.354) | 0.192 (0.039, 0.345) | 0.161 (-0.017, 0.339) |
|  | 16-18 (n=4035) | 0.164 (0.005, 0.323) | 0.171 (0.030, 0.312) | 0.177 (0.029, 0.325) |
| **Deprivation** | Most deprived  (n=2337) | 0.161 (0.003, 0.319) | -0.017 ( -0.125, 0.092) | -0.083 ( -0.185, 0.019) |
|  | Not most deprived  (n=4278) | 0.249 (0.119, 0.378) | 0.244 (0.108, 0.380) | 0.241 (0.096, 0.385) |
| **Year of Birth** | 1991-1994 (n=2443) | 0.041 ( -0.050, 0.132) | 0.011 (-0.107, 0.129) | -0.038 ( -0.152, 0.075) |
|  | 1995-1998(n=4172) | 0.220 (0.115, 0.326) | 0.214 (0.085, 0.343) | 0.221 (0.091, 0.350) |
| **History of SSRI prescription** | History (n=336) | 0.051 ( -0.306, 0.408) | -0.017 ( -0.235, 0.202) | 0.063 (-0.146 , 0.272) |
|  | No history n=6249) | 0.251 ( 0.120 , 0.381) | 0.272 ( 0.147 , 0.396) | 0.280 ( 0.150 , 0.410) |

**Note:** We adjusted for the following confounder: individuals’ sex, deprivation quintiles, year of birth, urban residence area, low birth weight status, age at earliest depression record, winter birth status, year of earliest depression record, history of inpatient CAMHS contact, population size of the general practices (GP) they were registered to, % of population per GP who belong to each of the five deprivation quintiles, % of population per GP with a CAMHS contact, % of population per GP who are from urban area. In each confounder strata the corresponding confounder was not adjusted for that analysis. Analysis accounted for cluster robust standard errors at the general practice level. Individuals who develop the outcome prior to the end of the treatment window were omitted from the analysis.

SSRI: Selective Serotonin Reuptake Inhibitors, IV: instrumental variable, CI: confidence intervals

### **Supplementary 4**

**S4.1** Descriptive statistics on the average and range population size covered by GP clusters of our analysis.

In our study sample we have n= 6,615 individuals with adolescent depression and n=224 GP clusters. The average (mean) and range (IQR) population size covered by these GP clusters are:

**mean 29.53 and IQR 16.**

IQR: interquartile range, GP: General Practice

**S4.2** Descriptive statistics on the length of time from earliest adolescent depression (symptom/diagnosis) to SSRI prescription in our sample.

The median time (measured in days) from first adolescent depression record to first SSRI prescription in the study sample (n=6615) was 250.5 days

### **Supplementary 5:** Study cohort timeline

|  | **Year of follow up** | | | | | | | | | | | | | | | | | | | |
| --- | --- | --- | --- | --- | --- | --- | --- | --- | --- | --- | --- | --- | --- | --- | --- | --- | --- | --- | --- | --- |
| **Birth year** | **2004** | **2005** | **2006** | **2007** | **2008** | **2009** | **2010** | **2011** | **2012** | **2013** | **2014** | **2015** | **2016** | **2017** | **2018** | **2019** | **2020** | **2021** | **2022** | **2023** |
|  |  |  |  |  |  |  |  |  |  |  |  |  |  |  |  |  |  |  |  |  |
| **1991** | 13 | 14 | 15 | 16 | 17 | 18 | 19 | 20 | 21 | 22 | 23 | 24 | 25 | 26 | 27 | 28 | 29 | 30 | 31 | 32 |
| **1992** |  | 13 | 14 | 15 | 16 | 17 | 18 | 19 | 20 | 21 | 22 | 23 | 24 | 25 | 26 | 27 | 28 | 29 | 30 | 31 |
| **1993** |  | | 13 | 14 | 15 | 16 | 17 | 18 | 19 | 20 | 21 | 22 | 23 | 24 | 25 | 26 | 27 | 28 | 29 | 30 |
| **1994** |  | | | 13 | 14 | 15 | 16 | 17 | 18 | 19 | 20 | 21 | 22 | 23 | 24 | 25 | 26 | 27 | 28 | 29 |
| **1995** |  | | | | 13 | 14 | 15 | 16 | 17 | 18 | 19 | 20 | 21 | 22 | 23 | 24 | 25 | 26 | 27 | 28 |
| **1996** |  | | | | | 13 | 14 | 15 | 16 | 17 | 18 | 19 | 20 | 21 | 22 | 23 | 24 | 25 | 26 | 27 |
| **1997** |  | | | | | | 13 | 14 | 15 | 16 | 17 | 18 | 19 | 20 | 21 | 22 | 23 | 24 | 25 | 26 |
| **1998** |  | | | | | | | 13 | 14 | 15 | 16 | 17 | 18 | 19 | 20 | 21 | 22 | 23 | 24 | 25 |
|  |  |  |  |  |  |  |  |  |  |  |  |  |  |  |  |  |  |  |  |  |
|  |  |  |  |  |  |  |  |  |  |  |  |  |  |  |  |  |  |  |  |  |
|  | Depression | | | | | | | | | |  |  |  |  |  |  |  |  |  | End  of |
|  |  | | | | | |  |  |  |  |  |  |  |  |  |  |  |  |  | Follow-up |

Table S5.1. Table with study cohort. Entry to the sampling frame was defined as the earliest date of depression record occurring between 13-17 (inclusive) years of age. The latest possible date for outcome follow-up was November 2023 (end of study), with a possible maximum age range of 25-32 years of age. Contact with Child and Adolescent Mental Health Services (CAMHS) (not shown here) was considered at any point from birth up to and including age17.

### **Supplementary 6:**

**Table S6.1** Regional level variation in SSRI prescribing patterns across general practice clusters based on the standardized SSRI cumulative treatment in those with adolescent depression.

.

|  |  | **Median** | **10^th^ percentile** | **90^th^ percentile** | **Difference** |
| --- | --- | --- | --- | --- | --- |
| **Intervention windows from first depression** | **One year** | 0.173 | 0.082 | 0.335 | 0.254 |
|  | **Two years** | 0.18 | 0.096 | 0.323 | 0.227 |
|  | **Three years** | 0.188 | 0.1 | 0.333 | 0.233 |
|  | **Four years** | 0.199 | 0.119 | 0.319 | 0.2 |

Individuals who develop the outcome prior to the end of the treatment window were omitted from the analysis

SSRI: Selective Serotonin Reuptake Inhibitors

### **Supplementary 7**: IV probit model with average marginal effects

From our IV probit analysis, we found no evidence of a relationship between SSRI treatment and subsequent risk of psychosis over the different intervention windows, which is in line with the estimates from the 2SLS model. As with the 2SLS model, we only report the estimates from the intervention windows where the first-stage regression meets the conventional rule of the thumb (F-statistic >10).

**Table S7.1** IV Probit model with average marginal effects reported

| **Providers preference for SSRI prescribing across general practice clusters**  **(IV instruments)** | **Non-affective psychosis** |
| --- | --- |
| **One year intervention window** | **[AME, 95% CIs]** |
| IV effect Probit model | 0.113 (-0.026, 0.251) |
| **Two years intervention window** |  |
| IV effect Probit model | 0.003 (-0.004, 0.011) |
| **Three years intervention window** |  |
| IV effect Probit model | 0.007 (-0.001, 0.014) |
|  |  |

**Note:** We adjusted for the following confounder: individuals’ sex, deprivation quintiles, year of birth, urban residence area, low birth weight status, age at earliest depression record, winter birth status, year of earliest depression record, history of inpatient CAMHS contact, history of SSRI prescription, population size of the general practices (GP) they were registered to, % of population per GP who belong to each of the five deprivation quintiles, % of population per GP with a CAMHS contact, % of population per GP who are from urban area. Analysis accounted for cluster robust standard errors at the general practice level. Individuals who develop the outcome prior to the end of the treatment window were omitted from the analysis.

SSRI: Selective Serotonin Reuptake Inhibitors, IV: instrumental variable, CI: confidence intervals, AME: Average Marginal Effect

### **Supplementary 8:** Subgroup analyses

In the subgroup analyses we considered treatment each SSRI alone: fluoxetine only, citalopram only, sertraline only. In the sertraline only and citalopram only subgroup analysis, we did not find a non-weak instrument in all intervention windows, and thus we do not report the IV estimates for these analyses.

**Table S8.1** Association between the IVs (over the course of different treatment windows) and cumulative prescription of sertraline or citalopram.

|  |  | **Sertraline** | **Citalopram** |
| --- | --- | --- | --- |
| **N** (n=6615) | With prescription^1^ | n=1640 | n=2090 |
|  | Without prescription^1^ | n=4975 | n=4525 |
| **F-statistic**  (First-stage regression)^2^ | One year intervention window | 0.544 | 7.33 |
|  | Two years intervention window | 1.652 | 2.87 |
|  | Three years intervention window | 3.013 | 0 |
|  | Four years intervention window | 4.878 | 0.009 |

**Note:**

^1^Individuals **with** or **without** sertraline or citalopram prescription within the four years of intervention window since the earliest depression

^2^ We adjusted for the following confounder: individuals’ sex, deprivation quintiles, year of birth, urban residence area, low birth weight status, age at earliest depression record, winter birth status, year of earliest depression record, history of SSRI prescription, history of inpatient CAMHS contact, population size of the general practices (GP) they were registered to, % of population per GP who belong to each of the five deprivation quintiles, % of population per GP with a CAMHS contact, % of population per GP who are from urban area. Analysis accounted for cluster robust standard errors at the general practice level. Individuals who develop the outcome prior to the end of the treatment window were omitted from the analysis.

IV: instrumental variable

For the fluoxetine-only subgroup analysis we found two non-weak instrument (two & three years of intervention windows). Below we report descriptive characteristics (Table S7.2), the variability of the instrument across intervention windows (Table S7.3) and the IV estimates (Table S7.4) for that analysis. From our IV fluoxetine-only subgroup analysis, we found no evidence of causal relationship of fluoxetine treatment on later psychosis risk in the two year and three-year intervention windows.

**Table S8.2.** 1991-1998 Birth cohort clinical and demographic characteristics in those with adolescent depression.

|  | | **Adolescent Depression** | | |
| --- | --- | --- | --- | --- |
| Characteristics | | Overall | Depression **with** fluoxetine prescription* | Depression **without** fluoxetine prescription* |
| **N** | | **6615** | 2472 | 4143 |
| **Sex** (n [%]) | Female | 4788(72.38%) | 1890(76.46%) | 2898(69.95%) |
|  | Male | 1826(27.6%) | 582(23.54%) | 1244(30.03%) |
|  | Missing | 1(0.02%) | 0 | 1(0.02%) |
| **Age at first depression** (mean [SD]) | | 16.21(1.25) | 16.36(1.15) | 16.12(1.29) |
| **Age at first fluoxetine prescription** (mean [SD]) | | - | 17.16(1.4) |  |
| **Outcome** | |  |  |  |
| Non affective psychosis (n [%]) | | 273 (4.13%) | 125(5.06%) | 148(3.57%) |

**Note: ***Individuals **with** or **without** fluoxetine prescription within the four years of intervention window since the earliest depression.

CAMHS: Child and Adolescent Mental Health Service, SD: Standard deviation.

**Table S8.3.** Variability in provider’s preference for fluoxetine prescribing across general practice based on the standardized fluoxetine treatment in those with adolescent depression.

|  |  | **Median** | **10^th^ percentile** | **90^th^ percentile** | **Difference** |
| --- | --- | --- | --- | --- | --- |
| **Intervention windows** | **One year** | 0.096 | 0.036 | 0.181 | 0.145 |
|  | **Two years** | 0.09 | 0.039 | 0.161 | 0.122 |
|  | **Three years** | 0.083 | 0.033 | 0.151 | 0.118 |
|  | **Four years** | 0.078 | 0.033 | 0.141 | 0.108 |

Individuals who develop the outcome prior to the end of the treatment window were omitted from the analysis

**Table S8.4.** Instrumental variable approach investigating the relationship between provider’s preference for fluoxetine prescribing and non-affective psychosis risk in those with adolescent depression using Two-Stage Least Squares regression (2SLS).

| **Providers’ preference for fluoxetine prescribing**  **across general practices**  **(IV instruments)** | **Non-affective psychosis** |
| --- | --- |
| **One year intervention window** |  |
| Number of outcomes | 215 |
| F-statistic (First stage regression) | 6.97 |
|  |  |
| **Two years intervention window** |  |
| Number of outcomes | 190 |
| F-statistic (First stage regression) | 12.524 |
| IV effect 2SLS [*β* coefficient, 95% CI] | -0.064 (-0.394, 0.267) |
|  |  |
| **Three years intervention window** |  |
| Number of outcomes | 162 |
| F-statistic (First stage regression) | 13.867 |
| IV effect 2SLS [*β* coefficient, 95% CI] | -0.059 (-0.452, 0.334) |
|  |  |
| **Four years intervention window** |  |
| Number of outcomes | 136 |
| F-statistic (First stage regression) | 7.29 |
|  |  |

**Note:** We adjusted for the following confounder: individuals’ sex, deprivation quintiles, year of birth, urban residence area, low birth weight status, age at earliest depression record, winter birth status, year of earliest depression record, history of SSRI prescription, history of inpatient CAMHS contact, population size of the general practices (GP) they were registered to, % of population per GP who belong to each of the five deprivation quintiles, % of population per GP with a CAMHS contact, % of population per GP who are from urban area. Analysis accounted for cluster robust standard errors at the general practice level. Individuals who develop the outcome prior to the end of the treatment window were omitted from the analysis.

SSRI: Selective Serotonin Reuptake Inhibitors, Two-Stage Least Squares regression (2SLS), IV: instrumental variable, CI: confidence intervals

### **References:**

1. ANGRIST JD, IMBENS GW, RUBIN DB. Identification of Causal Effects Using Instrumental Variables. Journal of the American Statistical Association. 1996;91:444-55.

2. LABRECQUE J, SWANSON SA. Understanding the Assumptions Underlying Instrumental Variable Analyses: a Brief Review of Falsification Strategies and Related Tools. Curr Epidemiol Rep. 2018;5:214-20.

3. BAIOCCHI M, CHENG J, SMALL DS. Instrumental variable methods for causal inference. Stat Med. 2014 Jun 15;33:2297-340.

4. HEALY C, O'HARE K, LÅNG U, et al. Does treatment of adolescent depression reduce risk of later psychosis: A quasi-experimental study of selective serotonin reuptake inhibitor treatment in a total population cohort. Eur Psychiatry. 2025 Jun 25;68:e82.

5. STAIGER D, STOCK JH. Instrumental Variables Regression with Weak Instruments. Econometrica. 1997;65:557-86.

6. IMBENS GW, ANGRIST JD. Identification and Estimation of Local Average Treatment Effects. Econometrica. 1994;62:467-75.
